# Heavy Traffic Can Be a Pain in the . . . Ear?: Vehicle Emissions Linked to Otitis Media

**Published:** 2006-09

**Authors:** Carol Potera

Traffic is a major source of air pollutants, and more studies are looking at the role of traffic-related air pollution in children’s health. Researchers report in this issue that young children exposed to higher levels of traffic pollution have a greater incidence of otitis media (middle ear infections) than those exposed to lower levels **[*EHP* 114:1414–1418; Brauer et al.]**. In 2002, the same team found that such pollution increased the risk for asthma and upper respiratory tract infections in young children. Now they focus on otitis media because upper respiratory tract infections often progress to ear infections, which are one of the leading reasons for visits to doctors and the use of antibiotics in childhood.

The new study involved approximately 3,700 Dutch children and 650 German children surveyed from birth to age 2 years. Researchers in those countries monitored the concentrations of three common traffic-related pollutants (nitrogen dioxide, particulate matter less than 2.5 μm in diameter, and elemental carbon) at 40 different sites in each country, then used those data to estimate exposures at each child’s residence. The levels of pollutants measured were similar in both countries and fell within a range commonly experienced by people living in industrialized nations. Information about doctor-diagnosed otitis media came from questionnaires answered by parents.

Both groups of children showed an increase in otitis media in association with greater traffic pollution exposure. By age 2, a third of the children had experienced otitis media at least once. The adjusted odds ratios of contracting otitis media associated with modest increases in exposure to the different air pollutants ranged from 1.09 to 1.24, and the risk of ear infections was similar for each of the three pollutants measured. Although environmental tobacco smoke has been linked to otitis media in studies by other researchers, exposure to this agent did not alter the associations between traffic pollution and otitis media observed in this study.

Otitis media has been estimated to cost the U.S. health care system $3–5 billion yearly. These findings, the first to link traffic pollution to otitis media, represent an additional consequence of air pollution. Protecting children from exposure to vehicular emissions—for example, by building major roadways away from residential zones, improving automobile emission standards, and driving less—may reduce the risk of otitis media.

## Figures and Tables

**Figure:**
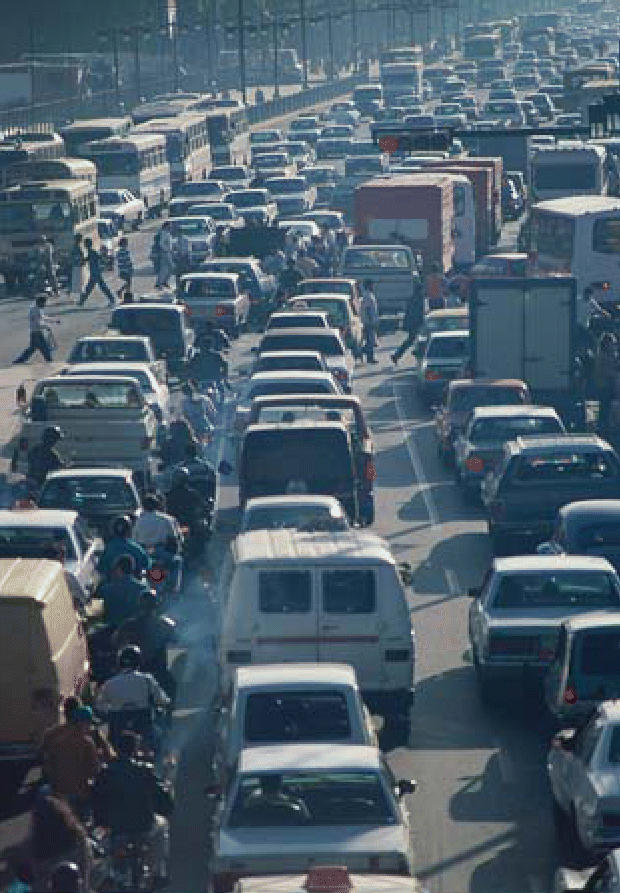
Aural pollution? Traffic pollution is linked to increases in cases of ear infection in children.

